# Editorial: Toward a New Understanding of LGBTAQ Youth: Promoting Innovative Perspectives

**DOI:** 10.3389/fpsyg.2020.544822

**Published:** 2020-10-09

**Authors:** Lilybeth Fontanesi, Angelo Brandelli Costa, Ugo Pace

**Affiliations:** ^1^Department of Psychological, Health and Territorial Sciences, University G.d'Annunzio of Chieti-Pescara, Chieti, Italy; ^2^Postgraduate Program in Psychology, Pontifical Catholic University of Rio Grande Do Sul, PortoAlegre, Brazil; ^3^Department of Human Sciences, University KORE of Enna, Enna, Italy

**Keywords:** LGBTAQ youth, sexual minorities, gender minorities, sexual development, gender development, sexual identities, gender identities and roles

People belonging to sexual and gender minorities are potentially exposed to situations of stigmatization and discrimination that negatively affect the construction of a positive social identity and social relationship. LGBTAQ (Lesbian, Gay, Bisexual, Trans, Asexual, Queer) people are frequently subject from an early age to minority stress and episodes of marginalization that may compromise self-perception and adaptation processes, in terms of attitudes, beliefs, feelings, and general well-being.

We proposed the Research Topic “Toward a New Understanding of LGBTAQ Youth: Promoting Innovative Perspectives” believing that a critical analysis of the needs of LGBTAQ people is already itself a practice of care and recognition, starting from childhood to early adulthood. Another reason underlying this Research Topic is that societies are so rapidly evolving that many differences may be found between and within generations in personal experiences and cultural expressions regarding sexuality and gender. Consequently, this Research Topic is meant to provide an up-to-date picture of a complex—still—stigmatizing society as well as the points on which it is necessary to focus to promote the health and guarantee the rights of LGBTAQ people.

The articles focused on childhood, adolescence, and young adulthood. Three main themes emerged from the papers: sexuality, homophobic victimization, and the psychological and social correlates, as well as their relationships, of gender identity and gender roles, in lesbian, gay, bisexual, and gender diverse young people. One of the main issues discussed is the lack of specific inclusive programs, whose development requires a more comprehensive evaluation of the strengths, personal and familial contexts, along with a clinical assessment of the needs of gender diverse young people. The issue was also encompassed by identifying risk factors (Pace et al.), the impact of stereotypes, stigma, and discrimination on sexuality among sexual and gender minorities as well as describing the relevance of identifying social, parental, and psychological contexts (Cerqueira-Santos et al.). Furthermore, it was possible to collect suggestions, insights, and clinical approaches to promote the best practice for psychologists, educators, and therapists working with sexual minorities, including genderqueer and non-binary youths (Scandurra et al.).

Finally, the articles analyzed the need for safe environments, where LGBTAQ children, adolescents, and young adults can freely express themselves, being accepted and included, building strong, positive self-concept and identity. Primary, secondary, and tertiary prevention were described as valuable instruments to implement adequate environments for these youths; when encompassing risk factors is essential to be able to promptly counteract bullying, discriminatory victimization, self-harm, and suicidal ideations.

We are thankful for all the researchers who shared their valuable work, and we believe that this Research Topic could be of inspiration and support to scholars, professionals, and readers who work with sexual and gender minorities.

## Author Contributions

All authors listed have made a substantial, direct and intellectual contribution to the work, and approved it for publication.

## Conflict of Interest

The authors declare that the research was conducted in the absence of any commercial or financial relationships that could be construed as a potential conflict of interest.

